# The effect of different methods and image analyzers on the results of the in vivo comet assay

**DOI:** 10.1186/s41021-017-0092-x

**Published:** 2018-02-07

**Authors:** Takahiro Kyoya, Rika Iwamoto, Yuko Shimanura, Megumi Terada, Shuichi Masuda

**Affiliations:** 1 Life Science Research Institute, Kumiai Chemical Industry Co., Ltd, 3360 Kamo, Kikugawa-shi, Shizuoka, 439-0031 Japan; 2 School of Food and Nutritional Sciences, University of Shizuoka, 52-1 Yada, Suruga-ku, Shizuoka, 422-8526 Japan

**Keywords:** Image analyzer, Comet assay IV, Comet analyzer, Validation, % tail DNA, EMS

## Abstract

**Introduction:**

The in vivo comet assay is a widely used genotoxicity test that can detect DNA damage in a range of organs. It is included in the Organisation for Economic Co-operation and Development Guidelines for the Testing of Chemicals. However, various protocols are still used for this assay, and several different image analyzers are used routinely to evaluate the results. Here, we verified a protocol that largely contributes to the equivalence of results, and we assessed the effect on the results when slides made from the same sample were analyzed using two different image analyzers (Comet Assay IV vs Comet Analyzer).

**Findings:**

Standardizing the agarose concentrations and DNA unwinding and electrophoresis times had a large impact on the equivalence of the results between the different methods used for the in vivo comet assay. In addition, there was some variation in the sensitivity of the two different image analyzers tested; however this variation was considered to be minor and became negligible when the test conditions were standardized between the two different methods.

**Conclusion:**

By standardizing the concentrations of low melting agarose and DNA unwinding and electrophoresis times between both methods used in the current study, the sensitivity to detect the genotoxicity of a positive control substance in the in vivo comet assay became generally comparable, independently of the image analyzer used. However, there may still be the possibility that other conditions, except for the three described here, could affect the reproducibility of the in vivo comet assay.

## Introduction

The single cell gel electrophoresis, or comet, assay is a genotoxicity test that, is able to detect DNA damage at the single-cell level [1]. It can be performed in vivo and in vitro and detects double and single strand breaks, alkaline labeling sites, oxidative-based damage, unrepaired DNA lesions and crosslink sites [2–5]. In vivo, it has the added advantage of detecting the organ-specific genotoxicity of chemicals.

The in vivo comet assay using rodents is widely recognized as a useful tool to assess genotoxicity; however, there is great variation in the protocol used [2]. This can have an important effect on the results of the assay [6], and a standardized method is needed to detect genotoxic substances with a high degree of accuracy and reproducibility. Therefore, based on the available literature, such a standard protocol was developed in Japan [4, 7–10]; it was used for the international validation of the in vivo comet assay. It was widely appreciated by the international scientific community and allowed the inclusion of the in vivo comet assay using rats in the Organisation for Economic Co-operation and Development (OECD) test guidelines (TG489). This standard protocol was adopted by a number of companies and contract research organizations. However, the choice of protocols used to perform the in vivo comet assay is decided by each institute. In addition, different image analyzers are used to evaluate the results of the comet assay [11–13].

Given this variability in the protocols and analyses used to perform the in vivo comet assay, it is of concern that the results for the same substance will change significantly among the different approaches used. Even within the same research institution, data for a given substance may be inconsistent if different protocols are used to obtain them. In this context, there is a case for the continued use of a specific and established protocol in order to guarantee the consistency of new and historical data obtained by each institute, thus allowing institutes to take advantage of their historical data.

It is necessary to understand which of the differences between the various protocols affect the results of the in vivo comet assay. Likewise, it is important to gauge the effects of the image analyzer used on the test results. Here, we therefore conducted two different protocols of the in vivo comet assay using tissue isolated from individual animals and investigated which method should be standardized to obtain equivalent results in different protocols. In addition, we investigated the effect of different protocols and image analyzers on the results of the in vivo comet assay.

## Materials and methods

### Materials

Ethyl methanesulfonate (EMS, CAS No. 62–50-0; Wako Pure Chemical Industries, Ltd., Tokyo, Japan) was used as a genotoxic positive control substance [14]. Physiological saline (PS) was used as a solvent (Otsuka Pharmaceutical Factory, Inc., Tokyo, Japan). Low melting agarose (LMA, NuSieve GTG) was purchased from Lonza (Basel, Switzerland), and standard melting agarose (SMA, Ultra Pure™ Agarose) was purchased from Thermo Fisher Scientific, Inc. (Massachusetts, USA). SYBR^Ⓡ^ gold was purchased from Thermo Fisher Scientific, Inc. (Massachusetts, USA). Ethidium bromide (EtBr) was purchased from Wako Pure Chemical Industries, Ltd. (Tokyo, Japan).

### Animals

Six week-old male Sprague-Dawley rats (Slc:SD) were supplied by Japan SLC, Inc. (Shizuoka, Japan) and acclimatized for 5 days prior to the experiments. Their age at the start of the experiment was 6–7 weeks. The rats were maintained on a 12 light/dark cycle at 24.5 ± 0.5 °C. They were fed a standard diet (CE-2; CLEA Japan, Inc., Tokyo, Japan) and were given tap water ad libitum.

### Treatment of animals

Rats in the treatment group (*N* = 4) were given 2 doses of EMS (200 mg/kg) in PS by oral gavage at 24 and 3 h before necropsy. Rats in the negative control group (*N* = 4) were treated in the same way, but given PS only.

### Image analyzers

A Comet Assay IV (Perceptive Instruments Ltd., Suffolk, UK) and a Comet Analyzer (Youworks Co., Tokyo, Japan) were used for image analysis. The parameter measured for comparison was % tail DNA. For measurements conducted using the Comet Analyzer, % tail DNA was calculated from the tail moment parameter, according to the manufacturer’s instructions.

We compared two different protocols for tissue preparation: the standard protocol of the international validation of the in vivo comet assay (Method 1) and a protocol based on a previously published paper (Method 2) [4]. To gain a better insight into which parameters of the protocols had the greatest impact on the results, we introduced a stepwise modification of Method 2 to bring it closer to Method 1. In Study 1, the original methods were used as reported [4, 10]. Next, we adjusted the LMA concentrations of Method 2 to those of Method 1 (Study 2). Finally, in Study 3, in addition to LMA concentration, we adjusted the DNA unwinding and electrophoresis times of Method 2 to match those of Method 1.

#### Tissue preparation for method 1 (standard protocol [10])

The rats were euthanized at 3 h after the second EMS or control treatment. Three or 4 cubes with an approximate side length of 5 mm were isolated from the left lateral lobe of the liver. The samples were minced in 3 mL cold mincing buffer of 20 mM ethylenediaminetetraacetic acid disodium salt (EDTA-2Na), 10% dimethyl sulfoxide (DMSO), and Hank’s balanced salt solution (Ca^2+^-and Mg^2+^-free) at pH 7.5 using dissection scissors. The samples were stored on ice for 30 s to allow large clumps to settle. Next, 10 μL of the supernatant were dispensed into a new 1.5 mL micro tube on ice. To this, 90 μL of 0.5% LMA in Dulbecco’s phosphate-buffered saline (DPBS) were added, and the mix was dripped onto a glass slide (Matsunami Grass Ind. Ltd., Osaka, Japan) coated with 1% SMA with DPBS. To facilitate an even spread of the mixture across the slide, it was covered quickly with another slide. After the agarose had solidified at 4 °C, the slides were separated, and another 90 μL of 0.5% LMA were added immediately in order to embed the cell layer fully in the agarose using another slide. In this way, 4 slides were prepared for each animal. After the top agarose layer had solidified, the slide was placed in a lysing solution of 2.5 M NaCl, 100 mM EDTA-2Na, 10 mM Tris-hydroxymethyl aminomethane (Tris), 1% Triton X-100, and 10% DMSO at pH 10 and left overnight in a cool and dark place. The slide was then rinsed with purified water and immersed in an alkaline solution of 300 mM NaOH and 1 mM EDTA-2Na for 20 min to unwind the DNA. Electrophoresis was carried out for 20 min at a voltage of 23 V (0.7 V/cm) and a current of 300 mA. Following this, the sample was neutralized by dripping an appropriate quantity of 400 mM Tris buffer (pH 7.5) onto the slide. After approximately 10 min, the slide was immersed in ethanol (≥99.6%) for more than 5 min to achieve dehydration. Approximately 60 μL SYBR Gold (Thermo Fisher Scientific, Massachusetts, USA), diluted 5000-fold with Tris/EDTA buffer (pH 8.0), were dripped onto the slide, which was then covered with a cover slip and observed through an epi-illumination fluorescence microscope. % tail DNA was measured for 50 randomly chosen cells per slide using the Comet Assay IV, and the median % tail DNA for 50 cells per slide was calculated. The average median for 2 slides was used as the DNA damage parameter for each animal. Cells with heads that were extremely small (% tail DNA ≥ 90%, “hedgehogs”) or irregularly shaped due to cytotoxicity, and which thus deviated substantially from the comet shape, were excluded from the analysis.

#### Tissue preparation for method 2 (adapted from [4])

Following the same procedure as for Method 1, 3 or 4 cubes with an approximate side length of 5 mm were isolated from the left lateral lobe of the liver. The samples were minced in 3 mL of cold mincing buffer of 30 mM EDTA-2Na and 0.9% (*w*/*v*) KCl at pH 7.5, using dissection scissors. Following this, the samples were stored on ice for 30 s to allow large clumps to settle. Next, 75 μL of the cell suspension were mixed with 75 μL 1.4% LMA in DPBS, and 75 μL of this mix (0.7% LMA) were dripped onto a slide coated with 0.7% SMA in DPBS, which was covered quickly with another slide to spread the mixture evenly across the slide. After the agarose had solidified at 4 °C, the slides were separated, and 75 μL of 0.7% LMA was dripped immediately onto the slide again to embed the cell layer fully in the agarose using another slide. Four slides were prepared for each animal. After the top layer of agarose had solidified, the slide was placed in a lysing solution (pH 10) of 2.5 M NaCl, 100 mM EDTA-2Na, 10 mM Tris, 1% sodium lauroyl sarcosinate (*w*/*v*), 1% Triton X-100, and 10% DMSO and left overnight in a cool and dark place. The next day, the slide was immersed in an alkaline solution of 300 mM NaOH and 1 mM EDTA-2Na for 10 min to unwind the DNA. Electrophoresis was carried out for 15 min at a voltage of 25 V (0.96 V/cm) and a current of 300 mA. Following this, the slide was neutralized by immersing it in 400 mM Tris buffer (pH 7.5) for approximately 10 min. Next, the slide was immersed in ethanol (≥99.6%) for more than 10 min to dehydrate the sample. Approximately 30 μL of 20 μg/mL EtBr was dripped onto the slide, which was then covered with a cover slip and observed through an epi-illumination fluorescence microscope. % tail DNA was measured for 50 randomly chosen cells per slide using the Comet Analyzer, and the median % tail DNA for 50 cells per slide was calculated. The average median for 2 slides was used as the DNA damage parameter for each animal. Cells with heads that were extremely small (% tail DNA ≥ 90%, “hedgehogs”) or irregularly shaped due to cytotoxicity, and which thus deviated substantially from the comet shape, were excluded from the analysis.

As indicated above, for Study 2, Method 2 was adjusted to match the LMA concentrations of Method 1. For Study 3, it was adjusted to match the LMA concentrations and DNA unwinding and electrophoresis times of Method 1 (see Table 1).

**Table 1:**
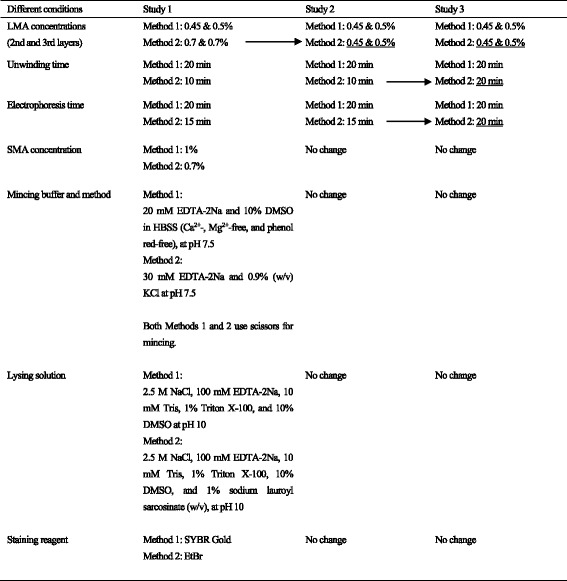
Differences in the conditions of Studies 1, 2, and 3

#### Study1: Comparison of the original methods and image analyzers

Methods 1 and 2 were conducted using tissue from the same animals. Four slides were prepared from each animal for each method, and two of the four slides were analyzed using each analyzer. The sensitivity of the two methods to detect the genotoxicity of a positive control substance (EMS) was evaluated by comparing the mean % tail DNA between the negative control and EMS-treatment groups for both analyzers.

#### Study 2: Matching of LMA concentrations

In this approach, the LMA concentrations used in Method 2 were adjusted to those used in the standard protocol (Method 1), i.e., 0.45% LMA was used for the second layer and 0.5% LMA was used for the third layer (see Table 1). All other parameters remained the same, and all comparisons were carried out as in Study 1.

#### Study 3: Matching LMA concentrations and DNA unwinding and electrophoresis times

The LMA concentrations and DNA unwinding and electrophoresis times used in Method 2 were adjusted to those used in Method 1 (see Table 1). All other parameters remained the same, and all comparisons were carried out as in Study 1.

### Statistical analysis

The median per each slide was calculated, and the average of these two median values was taken as mean % tail DNA per animal. For Methods 1 and 2, mean % tail DNA values, as measured by the Comet Assay IV or Comet Analyzer, were compared between the negative control and EMS-treatment groups. The homogeneity of the means was assessed using the *F*-test. Homoscedastic data were analyzed using a one sided Student’s *t*-test, and heteroscedastic data were analyzed with a one sided Aspin-Welch’s *t*-test.

## Results

**Fig. 1:**
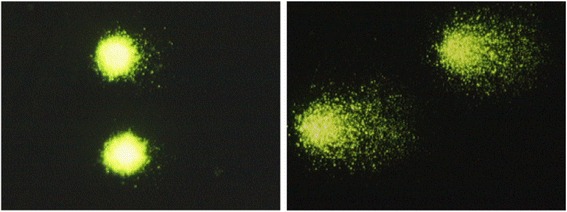
Typical comet images observed with SYBR Gold staining (Method 1)

**Fig. 2:**
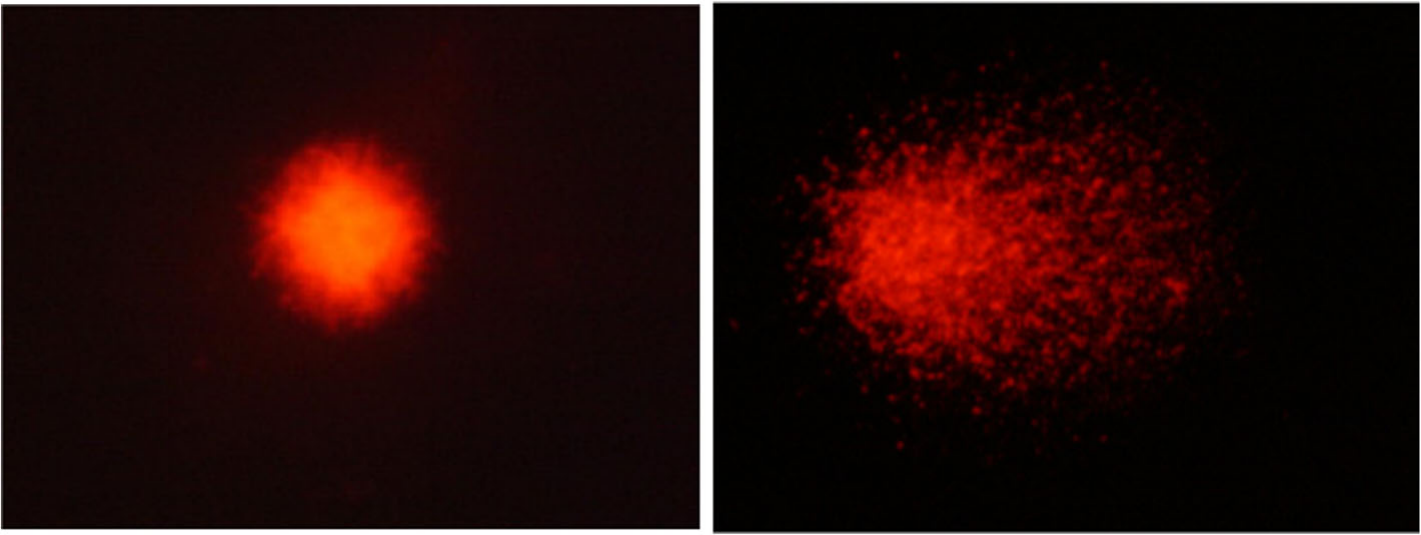
Typical Comet images observed with EtBr staining (Method 2)

All data are shown in Table 2. In all studies, the values of % tail DNA were significantly higher in the EMS-treatment group than in the PS negative control group. Typical comet images obtained using each method are presented in Figs. 1 and 2. The images on the left show typical results for the negative control group, while the ones on the right show typical results for the EMS-treatment group. In EMS-treatment group, DNA migration was observed using both methods, but the effect tended to be clearer with Method 1.

**Table 2:** Comet assay results for all studies

Protocol	Analyzer	Test substance	Mean % tail DNA ± SD		
			Study 1	Study 2	Study 3
Method 1	Comet Assay IV	PS	2.25 ± 0.58	1.48 ± 0.36	1.71 ± 0.76
		EMS	46.36 ± 5.78^##^	27.45 ± 11.89^#^	27.03 ± 1.41^**^
	Comet Analyzer	PS	4.04 ± 0.13	3.51 ± 0.42	1.34 ± 0.18
		EMS	35.12 ± 6.66^##^	18.54 ± 3.37^##^	20.40 ± 4.41^##^
Method 2	Comet Assay IV	PS	6.53 ± 4.34	3.39 ± 3.59	2.78 ± 1.16
		EMS	21.49 ± 13.05^*^	12.54 ± 5.47^*^	25.57 ± 7.92^##^
	Comet Analyzer	PS	4.19 ± 1.29	2.97 ± 0.52	4.37 ± 1.84
		EMS	8.56 ± 1.92^**^	11.05 ± 3.08^##^	15.34 ± 4.76^**^

Statistical analyses were conducted between the EMS-treatment and negative control groups for each of the method/analyzer combinations

*SD* standard deviation, *PS* physiological saline, *EMS* ethyl methanesulfonate

^*^p < 0.05, ^**^p < 0.01 (Student’s *t*-test)

#*p* < 0.05, ##*p* < 0.01 (Aspin-Welch’s *t*-test)

### Study 1

Study 1 compared the mean % tail DNA obtained using the two original methods. In Method 1 using the Comet Assay IV, the values of % tail DNA in the negative control and EMS-treatment groups were 2.25 and 46.36, respectively, an approximately 20.6-fold difference. For the Comet Analyzer, the values of % tail DNA in the negative control and EMS-treatment groups were 4.04 and 35.12, respectively, an approximately 8.7-fold difference.

In Method 2 using the Comet Assay IV, the values of % tail DNA in the negative control and EMS-treatment groups were 6.53 and 21.49, respectively, an approximately 3.3-fold difference. For the Comet Analyzer, the values of % tail DNA in the negative control and EMS-treatment groups were 4.19 and 8.56, respectively, an approximately 2-fold difference.

### Study 2

Study 2 compared the mean % tail DNA obtained when the LMA concentrations in Method 2 were standardized to those used in Method 1. In Method 1 using the Comet Assay IV, the values of % tail DNA in the negative control and the EMS-treatment groups were 1.48 and 27.45, respectively, an approximately 18.5-fold difference. For the Comet Analyzer, the values of % tail DNA in the negative control and EMS-treatment groups were 3.51 and 18.54, respectively, an approximately 5.3-fold difference.

In Method 2 using the Comet Assay IV, the values of % tail DNA in the negative control and EMS-treatment groups were 3.39 and 12.54, respectively, an approximately 3.7-fold difference. For the Comet Analyzer, the values of % tail DNA in the negative control and EMS-treatment groups were 2.97 and 11.05, respectively, an approximately 3.7-fold difference.

### Study 3

Study 3 compared the mean % tail DNA obtained when the LMA concentrations and DNA unwinding and electrophoresis times of Method 2 were standardized to those used in Method 1. In each group (treatment and control), there was a single slide from a single animal in which the cells had broken during analysis with the Comet assay IV, probably because the LMA had not solidified sufficiently. These slides were excluded from the analysis. As a result, the mean % tail DNA for the affected animals was calculated from a single slide (i.e., the median per one slide was used for the analysis) In Method 1 using the Comet Assay IV, the values of % tail DNA in the negative control and EMS-treatment groups were 1.71 and 27.03, respectively, an approximately 15.8-fold difference. For the Comet Analyzer, the values of % tail DNA in the negative control and EMS-treatment groups were 1.34 and 20.40, respectively, an approximately 15.2-fold difference.

In Method 2 using the Comet Assay IV, the values of % tail DNA in the negative control and EMS-treatment groups were 2.78 and 25.57, respectively, an approximately 9.2-fold difference. For the Comet Analyzer, the values of % tail DNA in the negative control and EMS-treatment groups were 4.37 and 15.34, respectively, an approximately 3.5-fold difference.

## Discussion

EMS is used as a positive genotoxic control in the standard protocol for the international validation of the in vivo comet assay and the OECD test guideline (TG489) [10, 14]. A statistically significant positive result for EMS is necessary to confirm the accuracy of the test. Here, the comparison of results between the EMS-treatment and negative control groups demonstrated that the mean % tail DNA was significantly higher in the treatment group than in the control group, irrespective of the method or image analyzer used. This confirms that both methods have the potential to detect genotoxic substances.

Study 1 found that, for the EMS-treatment group, the mean % tail DNA was lower for Method 2 than for Method 1, and the mean % tail DNA of the negative control group was relatively high. Thus, the difference was slight between the negative control and EMS-treatment groups in Method 2 using the Comet Analyzer. The mean % tail DNA values of all negative control groups, including the results of Studies 2 and 3, were within an acceptable range (1–8%) for the in vivo comet assay international validation standards [15]; however, the ratio of mean % tail DNA (3.3 and 2) between the negative control and EMS-treatment groups in Method 2 was much smaller than that in Method 1 (20.6 and 8.7). The ratio between the negative control and EMS-treatment groups was in the range of 2–18.5 for all of the participating institutes [15]. Its low sensitivity for the detection of positive substances was of concerned because there is a risk that the ratio of % tail DNA would be lower than 2 depending on the tests performed using Method 2. In contrast, the ratio between the negative control and EMS-treatment groups was 20.6 in data of the Comet assayIV in Method 1, which was higher than the above range; however, the absolute value of % tail DNA was 46.36, which was lower than the predicted maximum mean value of the EMS-treatment group (approximately 70 or more) in the in vivo comet assay international validation study [15]. In other words, this high absolute value was not abnormal, and the higher ratio was due to the low value of the negative control group; thus, it was considered that Method 1 had sufficient sensitivity to detect genotoxic substances.

This low sensitivity of Method 2 was likely due to the LMA concentrations and DNA unwinding and electrophoresis times. This is because when the concentration of LMA is high, it is difficult for DNA fragments to pass through the gel, and when the unwinding time is short, there is an insufficient amount of unwound double-stranded DNA, and the DNA migrates less easily due to the short electrophoresis time. The impact of these three parameters has already been evaluated for the in vitro comet assay, and an increase in % tail DNA values in response to decreased agarose concentrations and extended unwinding and electrophoresis times has been reported [16, 17]. From these results, in the case of a test substance such as acrylamide, for which a clear positive result is difficult to obtain using the in vivo comet assay, despite it being a known genotoxic carcinogen, it is of concern that the potential differences between the results of Methods 1 and 2 may occur due to this low sensitivity for a positive control substance as a consequence of unsuitable dose-settings [15, 18, 19]. Since the test substances was administered at the maximal tolerated dose in the in vivo comet assay, there was an upper limit to the dose. In order to maintain a certain detection sensitivity for a limited administration dose, the results of Studies 2 and 3 indicated that the LMA concentrations and DNA unwinding and electrophoresis times of Method 1 are appropriate.

An electrophoresis voltage of 0.7 to 1.0 V/cm has been recommended [4]. While a voltage outside the recommended range would likely affect the results, it is unlikely that variation within this range would have significant impact. Similarly, at a constant voltage, variation of the current between 200 to 400 mA is not likely to have meaningful effect on the results [17]. Our experiments showed very clearly that by standardizing the LMA concentrations and DNA unwinding and electrophoresis times, highly comparable results can be obtained from both methods without standardizing the remaining parameters of the in vivo comet assay. However, several other parameters varied between the two methods, including the SMA concentration used for the bottom layer of the slides, the composition of the mincing buffer and lysing solution, and the DNA staining method. It is likely that these parameters could affect the judgement of a positive result, with the exception of SMA, which was not in immediate contact with the cells. In Studies 1, 2, and 3, the mean % tail DNA values in the EMS-treatment group assessed using the Comet Analyzer tended to be low. The dark red fluorescence of EtBr was weaker than the fluorescence of SYBR Gold in microscopic observations, so it was suspected that this weak fluorescence might have affected the sensitivity to detect the tail of the comet. As it was not possible to clarify the effects of the different compositions of the mincing buffer and lysing solution on the results, it is likely that there is room for further research in the future. Therefore, in order to improve the detection sensitivity of genotoxic substances, it is considered preferable that these conditions should also be matched to those of Method 1 wherever possible.

Regarding the image analyzers, overall, the sensitivity of the Comet Assay IV seemed to be slightly higher than that of the Comet Analyzer from the results of this study. Especially, the mean % tail DNA (8.56%) of the EMS-treatment group was extremely low for Method 2 in Study 1. It was suggested that this low % tail DNA value could be improved by using SYBR Gold as the fluorescence stain. However, upon standardization of the experimental parameters in Study 3, the difference in the results obtained using the Comet Assay IV and Comet Analyzer was small, and the choice of image analyzer had little impact.

Thus, we conclude that, by standardizing LMA concentrations and DNA unwinding and electrophoresis times at least, DNA damage can be evaluated with equal sensitivity using different test protocols and image analyzers. While, in order to decrease the incidence of false negative results, we consider it desirable to conduct these tests using a method with high sensitivity for genotoxic substances, such as like the standard protocol for the in vivo comet assay international validation.

## Abbreviations

DPBS: Dulbecco's phosphate-buffered saline
EMS: Ethyl methanesulfonate
EtBr: Ethidium bromide
LMA: Low melting agarose gel
OECD: Organisation for Economic Co-operation and Development
PS: Physiological saline
SMA: Standard melting agarose gel
